# Dataset on null LC–MS/MS findings for 17 pharmaceuticals in Brazilian seafood with in-silico PBT/PMT indicators for monitoring design

**DOI:** 10.1016/j.dib.2025.112264

**Published:** 2025-11-11

**Authors:** Vinicius Roveri, Ursulla Pereira Souza, João Henrique Alliprandini da Costa, Jorge Luis dos Santos, Walber Toma, Simone dos Anjos Caivano, Alberto Teodorico Correia, Luciana Lopes Guimarães

**Affiliations:** a Universidade Metropolitana de Santos (UNIMES), Avenida Conselheiro Nébias, 536 - Encruzilhada, 11045-002 Santos, São Paulo, Brasil; b Centro Interdisciplinar de Investigação Marinha e Ambiental (CIIMAR/CIMAR), Avenida General Norton de Matos S/N, 4450-208 Matosinhos, Portugal; c Laboratório de Pesquisa em Produtos Naturais, Universidade Santa Cecília (UNISANTA), Rua Oswaldo Cruz, 266, C21, bloco C, 11045-907 Santos, São Paulo, Brasil; d Laboratório de Biologia de Organismos Marinhos e Costeiros (LABOMAC), Universidade Santa Cecília (UNISANTA), 11045-040 Santos, São Paulo, Brasil; e Programa de Pós-Graduação em Biodiversidade de Ambientes Costeiros, Universidade Estadual Paulista (UNESP), Campus Litoral Paulista, São Vicente, SP, Brasil; f Laboratório de Dietética Experimental^,^ Universidade Federal de São Paulo (UNIFESP), Campus Baixada Santista, 11030-100 Santos, São Paulo, Brasil; g Faculdade Israelita de Ciências da Saúde Albert Einstein (FICSAE), Rua Comendador Elias Jafet, 755, Morumbi, 05653-000, São Paulo, São Paulo, Brasil; h Instituto de Ciências Biomédicas Abel Salazar (ICBAS), Universidade do Porto (UP), Rua de Jorge Viterbo Ferreira 228, 4050-313 Porto, Portugal

**Keywords:** Null results, <LOD, LC–MS/MS, Brazilian seafood, Coastal monitoring, PBT, PMT, *In-silico* screening

## Abstract

Null results are rarely reported yet are essential to establish realistic baselines for marine–seafood contamination. This dataset documents the first attempt to detect 17 widely used pharmaceuticals in edible tissues (muscle and liver) of commercially important Brazilian coastal fish (*Centropomus parallelus, Cynoscion virescens, Nebris microps*) and shrimp (*Litopenaeus schmitti, Xiphopenaeus kroyeri*) collected near Perequê Island (Guarujá, São Paulo, Brazil). All analytes were below method detection limits (<LOD) by Liquid Chromatography coupled with Tandem Mass Spectrometry (LC–MS/MS), yielding a regionally relevant null-finding baseline for these targets at the time and place of sampling.

To add interpretative value and support reuse, we provide a complementary in-silico screening for Persistence (P), Bioaccumulation (B), Toxicity (T), and Mobility (M) properties (PBT/PMT framework). The Excel workbook assembles identifiers; fate properties (organic carbon–water partition coefficient: logKoc/Koc, bioconcentration factor: BCF/logBCF, environmental half-life, ready biodegradability); aquatic toxicity (acute median effective/lethal concentrations: EC50/LC50; chronic value: ChV across algae, crustaceans, and fish); and Sewage Treatment Plant (STP) removal (total, sludge adsorption, biodegradation), together with traffic-light flags and priority scores/ranks. Predictions were generated using fixed-version tools, namely OPEn structure–activity Relationship App (OPERA v2.8), Estimation Programs Interface Suite (EPI Suite v4.11; including KOCWIN v2.00, BCFBAF v3.01, BIOWIN v4.10, and wastewater treatment plant outputs), Ecological Structure–Activity Relationships (ECOSAR v2.0), and SimpleTreat 4.0, all of which are traceable via evidence fields and conditional formatting. The dataset enables (i) transparent publication of non-detects, (ii) prioritization of substances that may still warrant focused monitoring due to PBT/PMT attributes despite current non-detection, and (iii) benchmarking against future measurements or higher-tier assessments in urban–coastal waters.

## Graphical abstract

**Figure:**
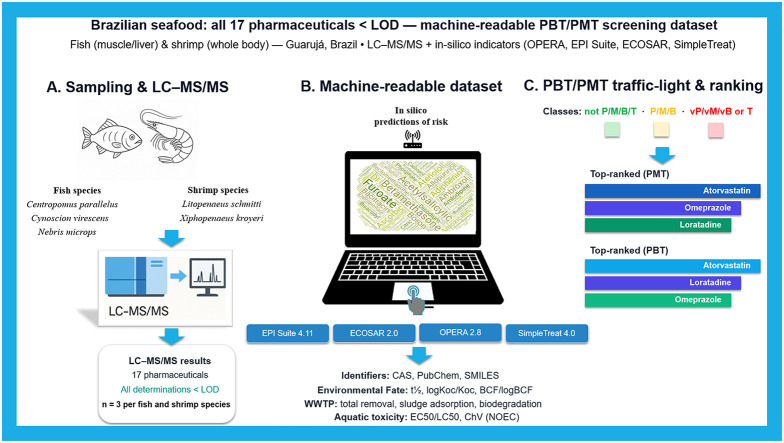

Specifications Table

**Table:** 

Subject	Earth & Environmental Sciences
Specific subject area	Screening dataset for PBT/PMT characterisation of pharmaceuticals
Type of data	Figures (.png); Tables (.xlsx). Processed and analysed data
Data collection	Tissue analyses were performed using an Agilent high-performance liquid chromatography system (HPLC; Agilent Technologies, CA, USA) coupled to a 3200 QTRAP hybrid triple quadrupole/linear ion trap mass spectrometer (AB Sciex, ON, Canada). In parallel, in-silico predictions were generated with OPERA v2.8, EPI Suite v4.11 (modules: KOCWIN v2.00, BCFBAF v3.01, BIOWIN v4.10, and WWTP outputs), ECOSAR v2.0, and SimpleTreat 4.0. All outputs were consolidated and curated into Excel workbooks, accompanied by evidence fields and traffic-light rules to support transparent interpretation.
Data source location	Fish and shrimp samples were collected in the coastal area adjacent to Perequê Island, Guarujá, São Paulo State, Brazil (23°55′53″S, 46°09′36″W to 23°55′11″S, 46°09′21″W). Laboratory analyses and *in-silico* predictions were performed at the Universidade Santa Cecília (UNISANTA) – Laboratório de Pesquisa em Produtos Naturais (LPPN) and Laboratório de Biologia de Organismos Marinhos e Costeiros (LABOMAC), Santos, São Paulo, Brazil
Data accessibility	Repository name: Mendeley Data Data identification number (DOI): 10.17632/p5jvw28tzj.1 Direct URL to data: https:// https://data.mendeley.com/datasets/p5jvw28tzj/1 Instructions for access: Openly accessible (public access via repository).
Related research article	None

## 1 Value of the Data

• Establishes a baseline of below-LOD findings for 17 pharmaceuticals in Brazilian coastal seafood (three fish and two shrimp species), addressing the common under-reporting of null results and enabling future meta-analyses and trend detection [[1], [2], [3], [4], [5], [6], [7], [8]]. Although all analytes were <LOD, this null finding also suggests, for the time and location of sampling, a favorable environmental quality status and a low immediate risk to public health through seafood consumption.
• Couples non-detects with reproducible *in-silico* PBT/PMT screening (P, M, T, B) using fixed-version tools—OPERA v2.8; EPI Suite v4.11 (KOCWIN v2.00, BCFBAF v3.01, BIOWIN v4.10, WWTP outputs); ECOSAR v2.0; SimpleTreat 4.0—so that prioritisation remains possible even when occurrence is zero or non-quantified [[4], [5], [6],^8^,^9^].
• Guides monitoring design and resource allocation: traffic-light coding and ranking scores highlight substances warranting targeted surveillance despite current non-detection; STP partitions (sludge adsorption vs. biodegradation) inform hypotheses on sources and attenuation processes.
• Maximises reusability through a machine-readable workbook, with sheet-level documentation and evidence fields that can be directly integrated into regulatory screening pipelines, mixture-risk dashboards, and fate/transport or LCA models.
• Enhances comparability and auditability: alignment with REACH PBT and emerging PMT frameworks, explicit provenance, and conditional formatting support transparent cross-study benchmarking and long-term updates [[1], [2], [3], [4], [5], [6], [7], [8]].

## 2 Background

Pharmaceuticals are increasingly reported in urban–estuarine waters worldwide, yet structured, reusable resources that combine instrument-ready LC–MS/MS outputs with screening-level persistence, mobility, and bioaccumulation flags remain scarce for tropical, highly urbanized coastal systems in Latin America [[10], [11], [12], [13], [14], [15], [16], [17], [18], [19]]. This dataset integrates (i) targeted LC–MS/MS screening outputs for 17 widely used pharmaceuticals spanning ten therapeutic classes, and (ii) harmonised *in-silico* indicators relevant to PMT/PBT screening (e.g., logKoc/Koc, BCF/logBCF, biodegradation readiness, and environmental half-life proxies) derived from widely adopted tools (OPERA, EPI Suite, BCFBAF, BIOWIN). The compilation is tailored to the Brazilian coastal system, characterized by wastewater inputs, tidal mixing, and short water-residence times [[10], [11], [12], [13], [14], [15], [16], [17]]. The files are standardized, annotated, and accompanied by method notes to facilitate reproducibility, cross-study comparability, and rapid triage for environmental assessments, regulatory screening, and teaching. All fields are defined and traceable to the specific prediction modules or analytical steps reported herein, enabling independent reuse and extension [5,8,9].

## 3 Data Description

The repository contains raw and processed screening outputs together with descriptive metadata and interpretation notes. Seventeen pharmaceutically active compounds spanning ten therapeutic classes were targeted: benzodiazepines (bromazepam [ATC: N05BA08], midazolam [ATC: N05CD08], diazepam [ATC: N05BA01], clonazepam [ATC: N03AE01]); SSRIs (fluoxetine [ATC: N06AB03], paroxetine [ATC: N06AB05]); H₂-receptor antagonist (ranitidine [ATC: A02BA02]); proton pump inhibitor (omeprazole [ATC: A02BC01]); PDE-5 inhibitors (tadalafil [ATC: G04BE08], sildenafil [ATC: G04BE03]); antihistamine – H₁ antagonist (loratadine [ATC: R06AX13]); atypical antipsychotic (risperidone [ATC: N05AX08]); antiandrogen/progestogen (cyproterone [ATC: G03HA01]); statins – HMG-CoA reductase inhibitors (simvastatin [ATC: C10AA01], atorvastatin [ATC: C10AA05], rosuvastatin [ATC: C10AA07]); and antiplatelet (P2Y₁₂ antagonist) (clopidogrel [ATC: B01AC04]).

Content overview (Tables S0–S10; Fig. 1, Fig. 2, Fig. 3, Fig. 4, Fig. 5, Fig. 6, Fig. 7, Fig. 8, Fig. 9, Fig. 10):

**Fig. 1:**
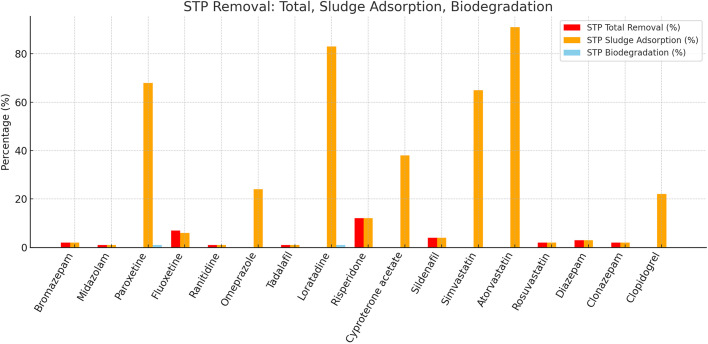
Sewage treatment plant (STP) removal of pharmaceuticals, showing contributions from total removal (red), sludge adsorption (orange), and biodegradation (blue).

**Fig. 2:**
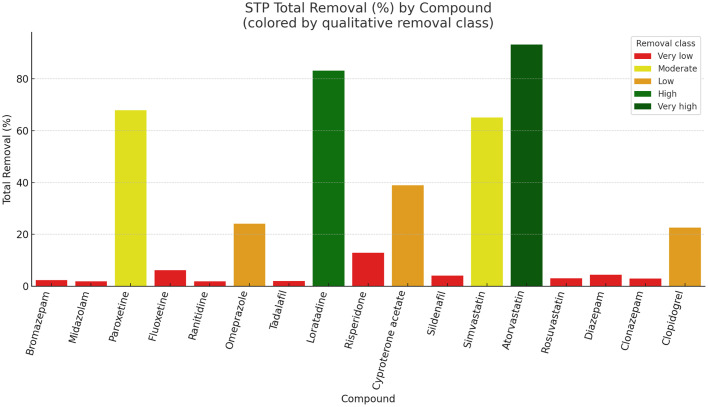
STP total removal ( %) of 17 target pharmaceuticals under an activated-sludge configuration. Bars are colour-coded according to removal class. Most compounds exhibited very low efficiencies, with only loratadine and atorvastatin reaching high to very high removal levels.

**Fig. 3:**
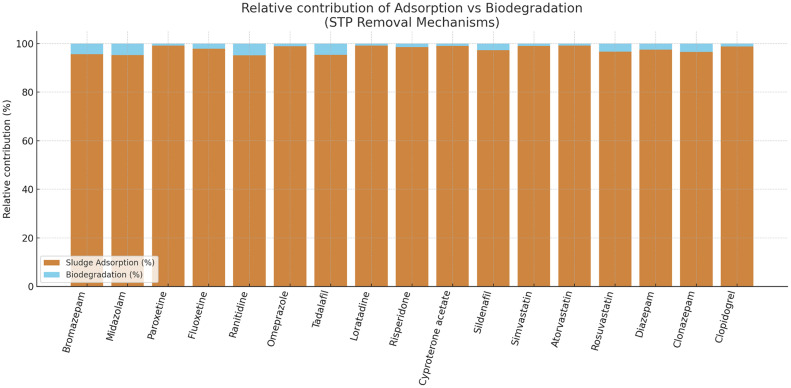
Relative contribution of sludge adsorption (brown) and biodegradation (blue) to the removal of 17 target pharmaceuticals. Removal was overwhelmingly dominated by adsorption, with biodegradation contributing <5 %.

**Fig. 4:**
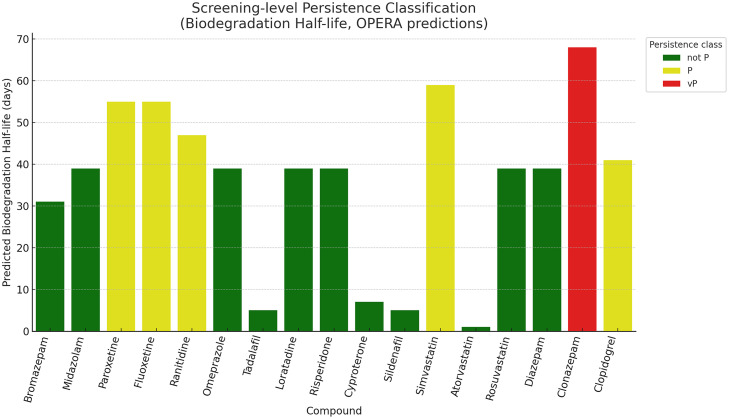
Screening-level persistence classification using OPERA-predicted half-life (days) and BIOWIN biodegradability flag. Most compounds were “not P”, while paroxetine, fluoxetine, ranitidine, simvastatin, and clopidogrel were persistent (P), and clonazepam was very persistent (vP).

**Fig. 5:**
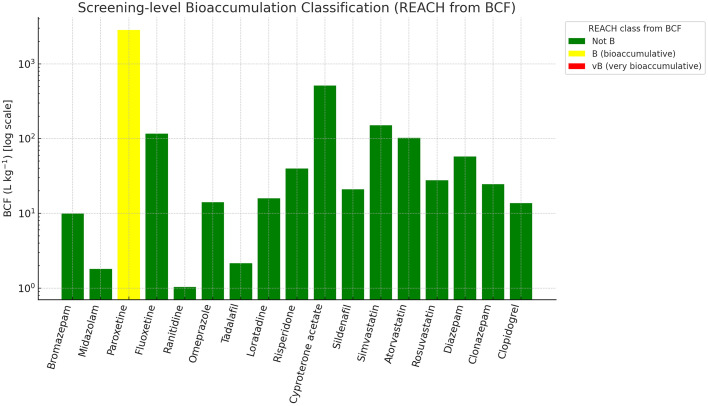
Bioaccumulation classification based on BCFBAF-predicted logBCF and BCF (L kg⁻¹). Paroxetine was classified as bioaccumulative (B), while all others were below thresholds.

**Fig. 6:**
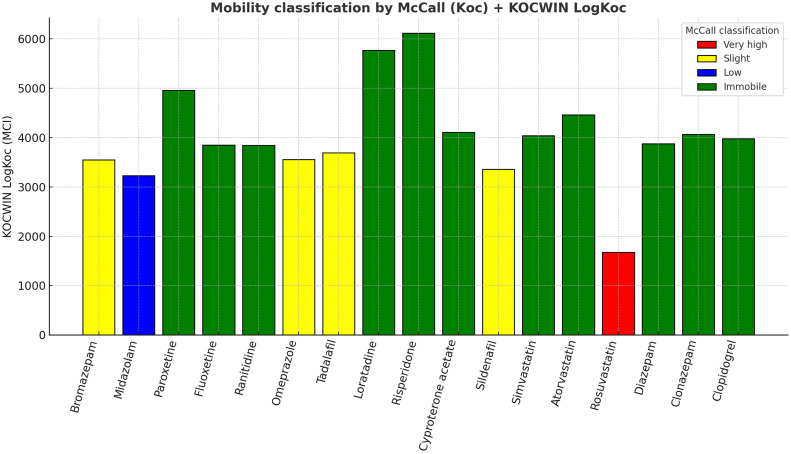
Mobility classification based on KOCWIN-predicted logKoc and estimated Koc, assigned to McCall categories: Very high, Slight, Low, or Immobile.

**Fig. 7:**
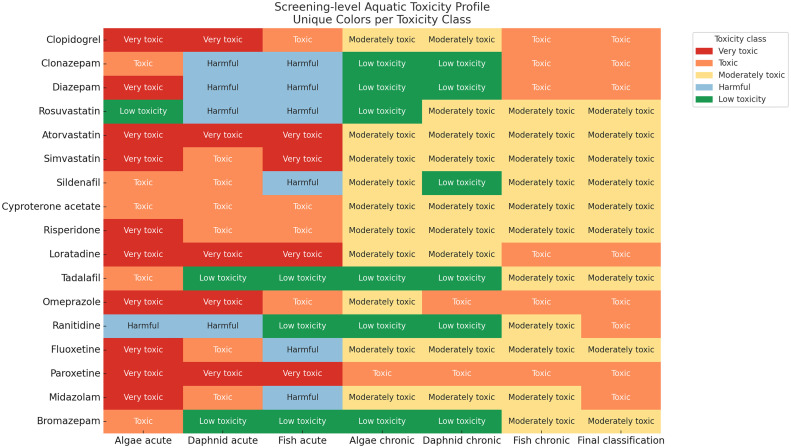
Aquatic toxicity profile including acute EC50/LC50 and chronic ChV values across algae, daphnids, and fish. Several compounds (e.g., paroxetine, fluoxetine, omeprazole, loratadine, simvastatin, atorvastatin, diazepam, clonazepam, clopidogrel) were in the toxic category.

**Fig. 8:**
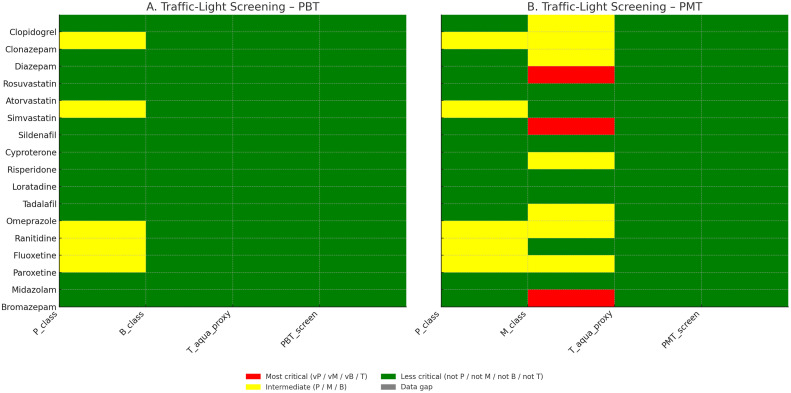
Traffic-light summary for PBT and PMT screening. No compound met overall thresholds, but several showed intermediate concern for persistence (e.g., paroxetine, fluoxetine, clonazepam, simvastatin) or mobility (e.g., sildenafil, rosuvastatin).

**Fig. 9:**
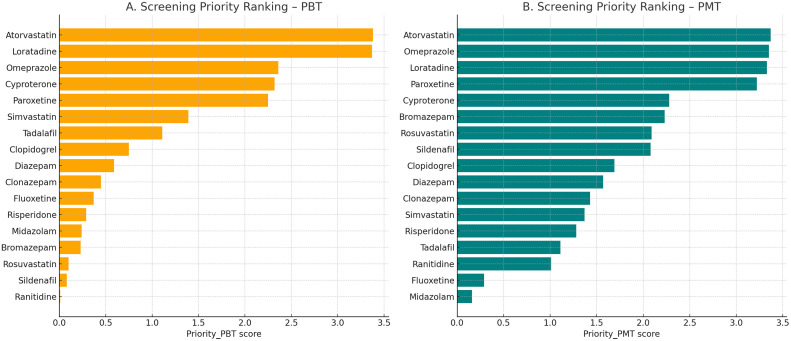
Screening priority rankings under PBT and PMT frameworks. Atorvastatin and loratadine ranked highest under PBT, while atorvastatin, omeprazole, and loratadine ranked highest under PMT.

**Fig. 10:**
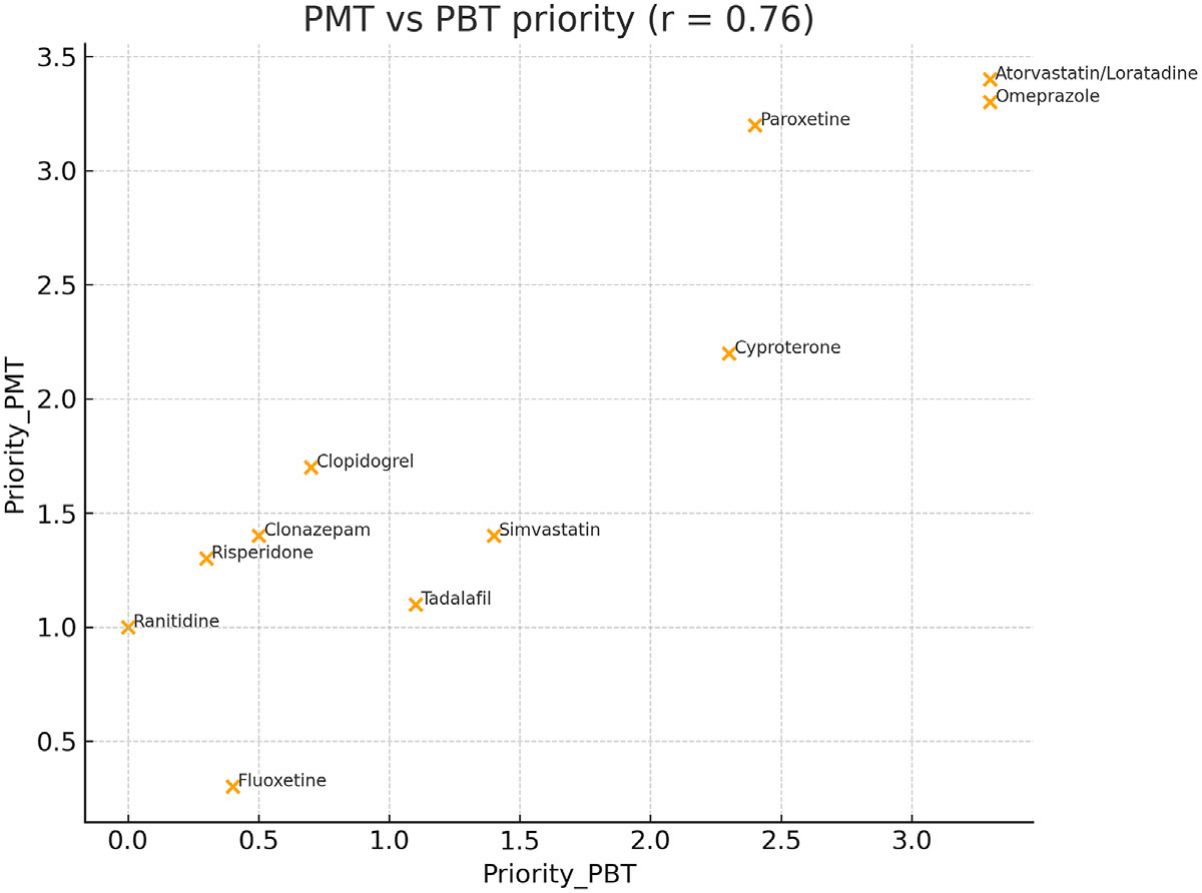
Correlation between PBT and PMT scores (*r* = 0.76). Substances prioritised under PBT also ranked highly under PMT; atorvastatin and loratadine displayed the highest combined scores.

Tables S0–S10:

(i) Compound-specific Multiple Reaction Monitoring (MRM) parameters, method limit of detection (MLOD), and retention times (RT) (S0).
(ii) LC–MS/MS null results for fish/shrimp tissues (S1).
(iii) Pharmacological context and identifiers (S2).
(iv) STP removal estimates (S3).
(v) Persistence screening (S4).
(vi) Bioaccumulation (S5).
(vii) Mobility (S6).
(viii) Aquatic toxicity endpoints (S7).
(ix) Traffic-light synthesis for PBT/PMT (S8).
(x) PMT priority scoring (S9.
(xi) Comparison of PBT vs PMT rankings (S10).

Together, these tables and figures provide a structured and reproducible framework linking null LC–MS/MS findings with in-silico fate and effect indicators. All variables, units, and classification rules are documented in the dataset, ensuring reproducibility, extension, and benchmarking.

## 4 Experimental Design, Materialsl and Methods

### 4.1 Materials and methods

#### 4.1.1 Study design overview

This dataset combines (i) an LC–MS/MS survey of 17 widely used pharmaceuticals in edible tissues of Brazilian coastal fish and shrimp, and (ii) a reproducible in-silico screening for PBT/PMT attributes of the same targets. Field samples were collected near Perequê Island (Guarujá, São Paulo, Brazil), processed and analysed by LC–MS/MS; all targets were below method detection limits (<LOD). To add interpretative value to these null findings, we generated screening indicators of persistence (P), mobility (M), bioaccumulation (B), and toxicity (T), with traffic-light coding and priority scores compiled in the accompanying workbook [8].

### 4.2 Fish and shrimp sampling sites

In June 2025 (austral winter), fish and shrimp were sampled by bottom trawling by local fishermen in the coastal area adjacent to Perequê Island, Guarujá, São Paulo State, Brazil (23°55′53″S, 46°09′36″W to 23°55′11″S, 46°09′21″W), and later donated for research purposes. Perequê Beach is the main artisanal fishing ground of the municipality, sustaining both subsistence and small-scale commercial fisheries [[12], [13], [14], [15]]. The area receives untreated urban discharges via four drainage channels that convey a complex mixture of contaminants—including nutrients, detergents, metals, pathogens, pharmaceuticals, and illicit drugs—into the Atlantic Ocean [[12], [13], [14], [15]]. The humid subtropical climate (annual rainfall of ∼3000 mm; mean temperature ∼22 °C) modulates pollutant inputs, with stronger runoff in November–March and reduced but continuous inputs in April–October [[12], [13], [14], [15]]. Three fish species (Centro*pomus parallelus, Cynoscion virescens, Nebris microps*) and two shrimp species (*Litopenaeus schmitti, Xiphopenaeus kroyeri*) were donated. Specimens were kept on ice (∼4 °C), transported to the laboratory, measured, dissected, and liver and muscle tissues were stored at −20 °C pending analysis.

### 4.3 Chemicals and standards

Seventeen pharmaceutically active compounds (PhACs) were prioritised based on (i) consumption in Brazil and (ii) toxicological relevance (observed or predicted) [[10], [11], [12], [13], [14], [15], [16], [17]]. Chromatography-grade acetonitrile, methanol, and isopropanol (Sigma-Aldrich, MA, USA) were used.

### 4.4 Tissue processing and extraction

Liver and muscle were separated; muscle was trimmed (∼2.5 cm cubes) and frozen at −20 °C. Prior to extraction, tissues were homogenised to fine powder (high-speed blender), following adjustments from Roveri et al. [18]. Approximately 1.0 g of homogenate was extracted with 8 mL of 0.1 M aqueous acetic acid:methanol (1:1, v/v), sonicated (15 min, 25 °C), and centrifuged (40 min, 3000 rpm). A 2 mL aliquot of supernatant was evaporated under nitrogen (45 °C), reconstituted in 0.2 mL water:acetonitrile (80:20, v/v), centrifuged (14,000 rpm, 3 min), and injected for LC–MS/MS.

### 4.5 LC–MS/MS analysis

Analyses were performed using an Agilent HPLC (Agilent Technologies, CA, USA) coupled to a 3200 QTRAP hybrid triple quadrupole/linear ion trap (AB Sciex, ON, Canada). An aliquot of 10 μL of each extract was injected. Separation was achieved on an Eclipse XDB-C18 (4.6 × 50 mm, 1.8 µm) column at 25 °C. Mobile phases: (i) Positive ESI: *A* = 0.1 % formic acid in water; *B* = acetonitrile. (ii) Negative ESI: *A* = 5 mM ammonium acetate buffer (pH 4.6); *B* = acetonitrile. A linear gradient (0.7 mL min⁻¹) was applied, from 95 % A and 5 % B to 5 % A over 5 min, held for 1 min, then re-equilibrated within 2 min. The ion spray voltage was set at 5500 V, and the source temperature at 650 °C. Detection was carried out in MRM with ESI ± polarity. Each analyte used one precursor and two product ions for quantification/confirmation. Data acquisition and integration were performed in Analyst® v1.5.2 (AB Sciex, Canada); no additional quantification software (e.g., Skyline) was used.

Each sample extract was injected in triplicate to verify instrumental precision.

The limit of detection (LOD) was defined as a signal-to-noise ratio (SNR) of 3:1.

Compound-specific MRM transitions, retention times, and method limits of detection (LOD) are presented in the Supplementary Table S0 (“Parameters of Multiple Reaction Monitoring, method limit of detection and retention time”), which will be available in the Mendeley Data repository.

Raw instrument files were not uploaded because all analytes were below detection limits (<LOD) in all injections. Baseline chromatograms were provided in the Mendeley Data repository for transparency and reproducibility.

### 4.6 Analytical QA/QC and handling of non-detects

Routine Quality Assurance / Quality Control (QA/QC) included solvent and procedural blanks and multi-point calibration. Across all biological extracts, target responses were below LOD under the conditions described; results are reported as non-detects (<LOD). Where both qualifier and quantifier transitions were triggered below LOD, results remained classified as non-detects. No background contamination above LOD was observed in blanks. Standard calibration curves were not provided because no compound was quantified above the detection limit, and isotopically labeled internal standards were not used.

### 4.7 *In-silico* property predictions and flags (PBT/PMT)

Screening-level PBT/PMT indicators were generated as follows: (i) Mobility (logKoc/Koc) with OPERA v2.8, cross-checked where needed with KOCWIN v2.00 within EPI Suite v4.11; (ii) Bioaccumulation (BCF/logBCF) with BCFBAF v3.01 (EPI Suite v4.11), corroborated with OPERA where applicable; (iii) Persistence using OPERA environmental half-life (days) and BIOWIN v4.10 (EPI Suite v4.11) readiness flags; (iv) Aquatic toxicity with ECOSAR v2.0 (acute EC50/LC50; chronic ChV for algae, crustaceans, and fish, retaining the lowest value per substance); (v) Sewage treatment removal with SimpleTreat 4.0 (primary) and EPI Suite WWTP output (partition details). PBT criteria followed REACH guidance (P/vP on half-life; B/vB on BCF; T based on acute/chronic thresholds), while PMT screening used vM/M cut-offs based on logKoc bands. All computations and flags were curated into the workbook, with dedicated evidence fields, conditional-format traffic lights, and prioritisation scores (PBT and PMT) computed as additive indices [[1], [2], [3], [4], [5], [6], [7], [8], [9]].

## Limitations

• The screening indicators are model-based and depend on the applicability domain and parameterisation of EPI Suite/BCFBAF/BIOWIN; uncertainty should be considered alongside any threshold-based flag.
• No temporal trend or seasonal representativeness is implied; the dataset is a structured snapshot for reuse, not a time series.
• Mixture effects and transformation products are not covered; flags refer to parent compounds only.
• STP removal figures (where provided) are screening-level and may differ from site-specific plant performance.
• The dataset is not a full risk assessment; users should derive PNECs and site-specific RQs as appropriate.
• All file formats, software versions, and cut-off values are documented; if tools are updated, users should re-run predictions to ensure strict version parity.

## Ethics Statement

The fish were caught and donated by fishermen for research purposes, with no sampling conducted by researchers.

## CRediT Author Statement

VR: Conceptualization; Methodology; Investigation; Formal analysis; Writing – original draft. UPS, JHAC and JLS: Selection of fish species; Planning of fish obtention and sampling; Investigation; Review & Editing. WT; SAC; and ATC: Formal analysis; Review & editing; LLG: Conceptualization and Methodology Writing; Investigation; Formal analysis; Review & Editing.

## Data Availability

Mendeley DataIn Silico Dataset of Environmental Fate and Ecotoxicity Parameters for 17 Pharmaceuticals (Original data)

## Appendix Supplementary materials

Image, application 1
